# *Desulfitobacterium elongatum* sp. nov. NIT-TF6 Isolated from Trichloroethene-Dechlorinating Culture with Formate

**DOI:** 10.3390/microorganisms13081863

**Published:** 2025-08-09

**Authors:** Udaratta Bhattacharjee, Ryuya Tomita, Li Xie, Naoko Yoshida

**Affiliations:** 1 Department of Civil and Environmental Engineering, Nagoya Institute of Technology (Nitech), Gokiso-Cho, Showa-Ku, Nagoya 466-8555, Japan; udarattb@srmist.edu.in (U.B.); r.tomita.523@nitech.jp (R.T.); 2 Department of Biotechnology, School of Bio Engineering, SRM-Institute of Science and Technology, Kattankulathur, Chennai 603203, India; 3 State Key Laboratory of Lake and Watershed Science for Water Security, Nanjing Institute of Geography and Limnology, Chinese Academy of Sciences, Nanjing 211135, China; xieli@niglas.ac.cn; 4 Department of Civil Engineering, Graduate School of Engineering, Nagoya University, Nagoya 464-8603, Japan

**Keywords:** *Desulfitobacterium*, reductive dehalogenase, L-lactate dehydrogenase

## Abstract

A strictly anaerobic bacterium denoted as strain NIT-TF6 of the genus *Desulfitobacterium* was isolated from a trichloroethene-dechlorinating culture with formate. Cells were straight rods of 1.6–6 µm long and 0.25–0.5 µm in diameter and used H_2_, lactate, pyruvate, and malate as electron donors and thiosulfate and Fe (III)-citrate as electron acceptors. The genome of strain NIT-TF6 was 4.8 Mbp in size and included nine 16S rRNA genes. Phylogenetic analysis based on 16S rRNA sequences showed that NIT-TF6 shared the highest sequence similarity (96.39%) with *Desulfitobacterium hafniense* DCB-2ᵀ, forming an independent clade in the phylogenetic tree. Digital DNA-DNA hybridization (dDDH) and average nucleotide identity (ANI) values between strain NIT-TF6 and other *Desulfitobacterium* species ranged from 15.9 to 16.9% and from 71.68 to 72.51%, respectively. These are well below the thresholds for species delineation. A distinguishing feature of strain NIT-TF6 was its possession of both L-lactate dehydrogenase (L-LDH) and D-lactate dehydrogenase (D-LDH), in contrast to other *Desulfitobacterium* strains that exclusively express D-LDH. Based on the dDDH and ANI results, combined with physiological, phylogenetic, morphological, biochemical, genomic, and metabolic iron-related characteristics, strain NIT-TF6 has been proposed as a novel species within the genus *Desulfitobacterium*. The name *Desulfitobacterium elongatum* sp. nov. has been proposed for this strain, with NIT-TF6ᵀ designated as the type strain.

## 1. Introduction

*Desulfitobacterium* belongs to the *Bacillota* and plays a key role in bioremediation by using organohalides such as chlorinated ethenes (CEs), chloroethanes, and chlorinated phenolic compounds. It consists of three validated species, that is, *D. hafniense* DCB-2^T^ [1], *D. dehalogenans* JW/IU-DC1^T^ [2], *D. metallireducens* 853-15^T^ [3], and the yet-to-be validated “*D. dichloroelimans* DCA1” [4]. The genus also previously included *D. aromaticivorans*. However, this species has been reclassified as *Paradesulfitobacterium aromaticivorans* [5]. In terms of their valid species, *D. chlororespirans* DSM 11544 is a scaffold genome, and *D. frappieri* ATCC 700357 has been merged under the first described name, namely, *D. hafniense*. *Desulfitobacterium* species have been isolated from municipal sludge, methanogenic lake sediment, compost soil, uranium-contaminated aquifer sediment, and the soil matrix of an anoxic water-saturated layer, respectively. They are strictly anaerobic, rod-shaped, and spore-forming bacteria that have versatile energy conversions, including fermentation and respiration using a wide range of electron donors and acceptors. These valid strains confirmed their heterotrophic growth by gaining energy through versatile respiration with Fe (III) and nitrate, coupling with hydrogen or organic acids.

Recently, the genus has been receiving considerable attention in supporting *Dehalococcoides*, a representative organohalide-respiring bacterium that can fully dechlorinate trichloroethene (TCE) to non-toxic ethene [6,7,8]. In our previous study, a microcosm (FOR) of groundwater supplemented with formate was reported to dechlorinate TCE to ethene efficiently in the augmentation of *Dehalococcoides*. The FOR includes *Dehalococcoides*, *Methanosphaerula*, *Rectinema*, and *Desulfitobacterium*. It showed more rapid and efficient dechlorination compared to microcosms supplemented with other C2- and C3-organic acids, and absence of *Desulfitobacterium* [9]. *Dehalococcoides* use H_2_ and acetate as the sole electron donor and carbon source, respectively, and require cobalamin as a cofactor [10,11,12]. These are generally supplied by heterotrophs fermenting organic matter in the environment. *Desulfitobacterium* possesses genes for formate hydrogenlyase, which converts formate into H_2_ and CO_2_ [13]. It also produces cobalamin for *Dehalococcoides* [14] and carries the complete genome for the acetyl-CoA (Wood–Ljungdahl) pathway. This can convert CO_2_ into acetate via an autotrophic pathway through formate as an intermediate [13,15].

So far, the contributions of *Desulfitobacterium*, particularly in supporting TCE-to-ethene dechlorination via formate use, remain unclear. Therefore, further research is required. In this study, a novel strain NIT-TF6 of *Desulfitobacterium* was isolated from the FOR culture, and its potential role was clarified using physiological and whole-genomic analysis of the strain. It was compared with *D. hafniense* [1], *D. dehalogenans* [2], *D. metallireducens* [3], and “*D. dichloroeliminans*” [4].

## 2. Materials and Methods

### 2.1. Isolation and Growth Conditions

*Desulfitobacterium elongatum* NIT-TF6 was isolated from a FOR culture containing *Dehalococcoides mccartyi* NIT01. This was originally obtained from an enriched culture designated YN3 [16]. The inoculum used to prepare the YN3 culture was sediment from the Arako River, Nagoya, Japan [17]. The DS-basal medium used for isolating and cultivating the strain consisted of 20 g/L NaCl, 0.3 g/L KCl, 0.5 g/L NH_4_Cl, 0.1 g/L CaCl_2_·2H_2_O, 4 g/L MgCl_2_·6H_2_O, 0.6 g/L KH_2_PO_4_, 2.5 g/L NaHCO_3_, 1 mL/L SL-10, 1 mL/L Se/W solution, vitamin solution (per L consisted of 20 mg D-biotin, 20 mg folic acid, 100 mg pyridoxine-HCl, 50 mg vitamin B_1_ hydrochloride, 50 mg riboflavin, 50 mg nicotinic acid, 50 mg D-panthothenic acid calcium salt, 75 mg vitamin B_12_, 50 mg p-aminobenzoic acid, 50 mg nicotinamide, 50 mg lipoic acid, 50 mg hemin and 50 mg 1, 4-naphthoquinone), and 0.2 mg/L resazurin (all purchased from and manufactured in Japan). The basal medium was prepared anaerobically by flushing with a N_2_:CO_2_ gas mixture (80:20, *v*/*v*) [16]. NIT-TF6 was isolated using an anaerobic DS-FT agar-shake medium that comprises DS-basal medium supplemented with 10 mM lactate and 10 mM thiosulfate, and 1.5% agarose. After incubating for 7–14 d at 28 °C, the colony was purified through repeated agar-shake cultivation and re-cultivated in liquid DHB-CO3-LT medium. This comprises DHB-CO3 medium supplemented with 10 mM lactate and 10 mM thiosulfate. The purified culture was phylogenetically identified based on the 16S rRNA gene sequence amplified from cell lysates [18] and designated as strain NIT-TF6. Routine cultivation of NIT-TF6 was performed in liquid DHB-CO3-LT medium, with 7–14 d of anaerobic incubation at 28 °C required for complete growth. The source of the reference strains *D. dehalogenans* ATCC 51507^T^, “*D. dichloroeliminans*” DCA1, *D. hafniense* DCB-2^T^, *D. metallireducens* DSM 15288^T^ were methanogenic lake sediment, the soil matrix of a 1 m deep, anoxic, water-saturated layer that had been continuously contaminated with 1,2-dichloroethane for 30 years beneath an industrial storage tank, municipal sludge, and uranium-contaminated aquifer sediment.

### 2.2. Physiological, Morphological, and Biochemical Analyses

Acetate, fumarate, pyruvate, lactate, propionate, butyrate, methanol, ethanol, isopropanol, benzoate (all at 10 mM), and H_2_+acetate (6% *v*/*v* and 10 mM) were tested as potential electron donors for thiosulfate reduction. The suitability of various electron acceptors was evaluated by monitoring growth and detecting the oxidation of 10 mM pyruvate in the presence of thiosulfate, nitrate, nitrite, Fe (III) citrate, and air (1%) [19]. The morphology of *Desulfitobacterium elongatum* was examined using scanning electron microscopy (JSM-7800F; JEOL Ltd., Tokyo, Japan) at an operating voltage of 1.0 kV [19]. Gram-staining data were procured from Rapid Annotation using Subsystem Technology (RAST) server (https://rast.nmpdr.org/, accessed on 9 February 2025).

### 2.3. Sanger Sequencing of 16S rRNA Gene and Genome Pyrosequencing

Genomic DNA was extracted from *Desulfitobacterium elongatum*, and sanger sequencing was performed [17]. The sequencing was conducted using the Illumina MiSeq and Nanopore MinION platforms. A total of 1.8 million Illumina paired-end reads (1.02 Gbp, 150 bp × 2) and 0.37 million Nanopore reads (1.76 Gbp) were processed for error correction using Short Read Manager and assembled with Unicycler (v0.4.7, https://github.com/rrwick/Unicycler/releases/tag/v0.4.7, accessed on 9 February 2025). The complete genome of NIT-TF6 was successfully assembled, and gene prediction and annotation were performed using DFAST (https://dfast.ddbj.nig.ac.jp/, accessed on 9 February 2025) [20]. Comparative genomic analysis between NIT-TF6 and other *Desulfitobacterium* species was conducted using bidirectional best hits with a threshold of 40% identity and 80% query coverage, RAST (Rapid Annotation using Subsystem Technology) (http://rast.nmpdr.org/rast.cgi, accessed on 9 February 2025) [21], and the BLAST 2.17.0 (http://blast.ncbi.nlm.nih.gov/Blast.cgi, accessed on 9 February 2025) tool from the NCBI database. The complete genome sequence of strain NIT-TF6 was deposited in DDBJ/GenBank under the BioProject number PRJDB19082 with accession number AP038772 (BioSample identifier: SAMD00830004).

### 2.4. Phylogenetic Analysis Based on 16S rRNA Gene and Core Genome

The full-length 16S rRNA gene sequence of *Desulfitobacterium elongatum* was extracted from the complete genome data. Those for all valid strains of *Desulfitobacterium* spp. were downloaded from the RAST database. Phylogenetic analyses were performed using MEGA X (v8, Max Planck Institute of Biochemistry (MPIB), Planegg, Germany) (https://www.megasoftware.net/, accessed on 9 February 2025) based on the neighbor-joining method and maximum composite likelihood model [22]. Statistical support for the branches of the phylogenetic trees was determined using bootstrap analysis based on 1000 resamplings [23]. Digital DNA-DNA hybridization (dDDH) and ANI have replaced traditional DDH as the gold standard in prokaryote taxonomy for defining new species [24,25,26]. The dDDH and ANI values were calculated using Genome-to-Genome Distance version 3.0 [27] and the ANI calculator on the EzBioCloud platform [25].

### 2.5. TB Tool II for Genome Analysis

TBtools-II (v1.106) from the Kyoto Encyclopedia of Genes and Genomes (KEGG) framework was used to construct a circular genome of *Desulfitobacterium elongatum* (v1.106, available at https://github.com/CJ-Chen/TBtools-Manual, accessed on 9 February 2025).

### 2.6. GC-FID Analysis for Dechlorination Experiments

Volatile compounds such as TCE, *cis*-DCE, VC, ethene, and methane were analyzed by injecting the headspace gas into a gas chromatograph (model GC-2014, Shimadzu, Kyoto, Japan) equipped with a Porapak Q column (GL Sciences, Tokyo, Japan) and a flame ionization detector (FID) [17,18].

### 2.7. GC-MS for Detection of ^13^C-Labeled Acetate

^13^C-labeled acetate was detected using Gas Chromatography–Mass Spectrometry (GC-MS). For this, a 1 mL sample solution was mixed with 4 mL ethyl acetate, and the separate solvent was recovered by the addition of anhydrous sodium sulfate. Helium was considered a carrier gas with splitless injection. The column oven and injection temperature were maintained at 50 °C and 280 °C, respectively. The column oven temperature increased to 80 °C [28].

## 3. Results and Discussion

### 3.1. Isolation and Morphology of Strain NIT-TF6

**Figure 1:**
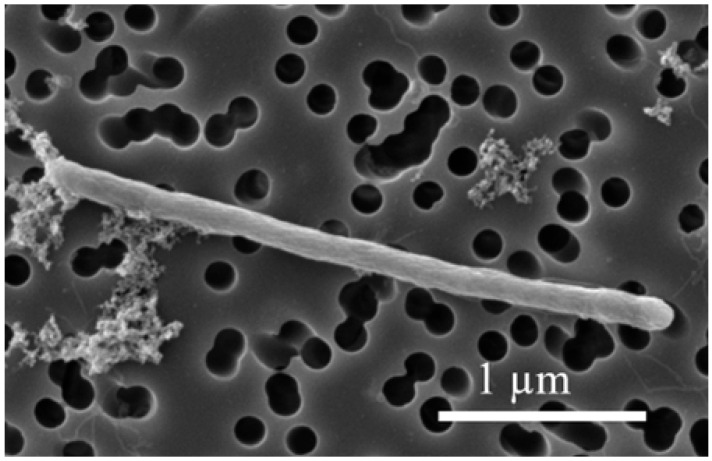
Scanning electron microscopic images of spore-forming strain NIT-TF6 in the exponential phase.

*Desulfitobacterium elongatum* NIT-TF6 was isolated using deep agar cultivation supplemented with 10 mM lactate and 10 mM thiosulfate from the FOR microcosm. The isolated strain includes long, straight rod-shaped cells measuring 1.6–6 µm in length and 0.25–0.5 µm in diameter (Figure 1).

### 3.2. Physiological Characteristics of Strain NIT-TF6

*Desulfitobacterium elongatum* NIT-TF6 can use various substrates, such as pyruvate, lactate, and H_2_+acetate, with thiosulfate as the electron acceptor (Table 1). Strain NIT-TF6 did not grow on thiosulfate respiration with a combination of acetate, fumarate, propionate, butyrate, methanol, ethanol, isopropanol, and benzoate. The substrates were chosen to represent a range of potential carbon sources and electron donors/acceptors that are relevant to the strain’s ecological niche. The selection was based on prior studies of related *Desulfitobacterium* species. Therefore, these substrates can cover the representative substrates that the strain can utilize.

### 3.3. Phylogenetic Analysis

**Figure 2:**
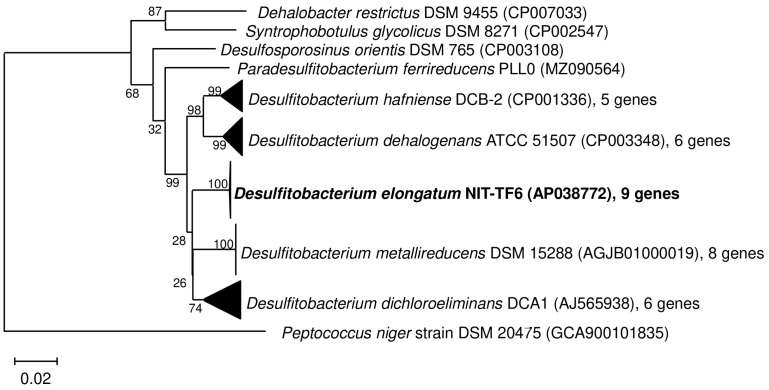
16S rRNA gene sequence-based neighbor-joining phylogenetic tree of the strain NIT-TF6 and its close relatives, namely type strains within the *Desulfitobacteriaceae* family. Bar indicates 0.02 substitutions per nucleotide position.

*Desulfitobacterium elongatum* NIT-TF6 shared the highest sequence similarity with *D. hafniense* DCB-2^T^ (96%) and formed a cluster with the closest relative and *D. metallireducens* DSM 15288^T^, *D. dehalogenans* ATCC 51507^T,^ and “*D. dichloroeliminans*” DCA1. Except for the mentioned genera, strain NIT-TF6 had the highest sequence similarity with *Desulfosporosinus orientis* DSM 765^T^ (94%). Based on these findings, strain NIT-TF6 likely represents a novel species within the family *Desulfitobacteriaceae* (Figure 2). A phylogenetic tree based on the whole genome of the strain NIT-TF6 and its close relatives, type strains within the *Desulfitobacteriaceae* family was considered based on TYGS (https://tygs.dsmz.de/, accessed on 7 January 2025) results (Figure S1—Supplementary Materials). However, the constructed phylogenetic tree is interchangeable within the species and subspecies level and does not necessarily represent similar data analyzed by 16S rRNA. This was clarified using dDDH and ANI analysis to identify species delineation.

### 3.4. General Genomic Feature

*Desulfitobacterium elongatum* NIT-TF6 contains a single circular chromosome consisting of 4,807,969 base pairs, 4655 protein-coding sequences (CDS), and 119 RNA sequences. Its G+C content is approximately 43.2 mol% and in the range observed in other *Desulfitobacterium* species (41.9–47.5%). Other genomic characteristics, such as genome size and CDSs, were consistent in the range of 3.1–5.2 Mb and 3138–5247, respectively, with previously reported values. However, strain NIT-TF6 contains nine copies of the 16S rRNA gene, which is higher than the typical range (5–8) reported in other *Desulfitobacterium* species (Table 2).

**Figure 3:**
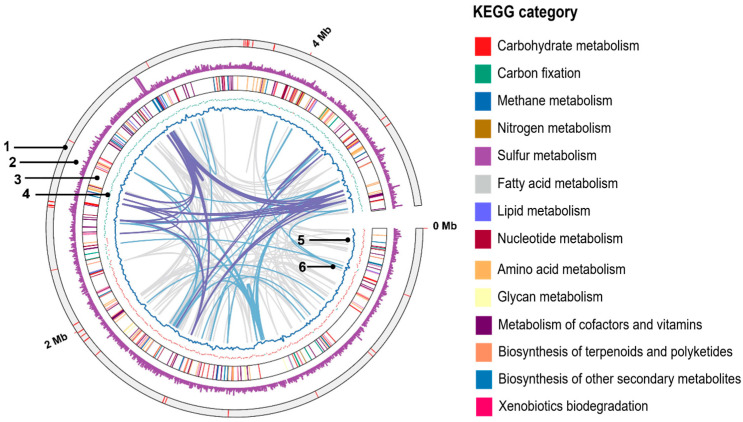
Circular genome map of *Desulfitobacterium elongatum* NIT-TF6 illustrated with a circular representation generated using TBtools-II. The rings are numbered sequentially from the outside to the inside as follows: 1, location of c-type cytochromes; 2, gene density; 3, protein-coding sequences colored based on KEGG category; 4, G+C skew (red, positive; green, negative); 5, G+C content; 6, links showing repetitive sequence ≥95% identity (pink, >500 bp; purple, >2 kbp). KEGG, Kyoto Encyclopedia of Genes and Genomes.

The recommended thresholds for classification of prokaryotic novel species are 60–70% for dDDH and 95–96% for ANI [29]. In this study, dDDH and ANI values between the strain NIT-TF6 and other *Desulfitobacterium* species were 15.9–16.9% and 71.68–72.51%, respectively. These are substantially below the widely accepted species threshold for identification. To further examine the functional annotation of genes associated with specific categories, a circular genome map of NIT-TF6 was constructed using TBtools-II (Figure 3). The highest gene numbers were associated with the metabolism of amino acids and their derivatives (148 genes). The amino acid metabolism dominance implicates that strain NIT-TF6 could sustain in an environment where amino acid is the primary energy source. This was followed by genes involved in carbohydrate metabolism (130 genes) that could support the growth phase of strain NIT-TF6 during nutrient-limiting conditions. Strain NIT-TF6 possesses genes related to the metabolism of cofactors and vitamins (80 genes) and energy metabolism (74 genes).

### 3.5. Metabolic Potential and Unique Characteristics Based on the Genome

**Figure 4:**
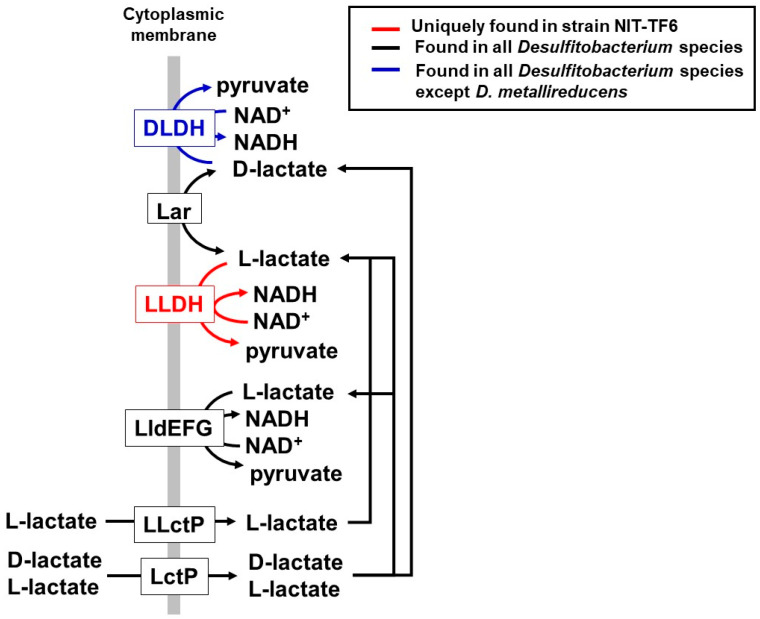
Lactate metabolism-related genes found in the genome of NIT-TF6 and other *Desulfitobacterium* species. L-lactate dehydrogenase (L-LDH, EC: 1.1.1.27) is only found in *Desulfitobacterium elongatum* NIT-TF6; L-lactate permease (L-LctP, T.C.: 2.A.14.1.1), lactate permease (LctP, T.C.: 2.A.14), lactate racemase (Lar, EC: 5.1.2.1), and L-lactate dehydrogenase complex (LldEFG, no EC number) are found in all *Desulfitobacterium* species. D-LDH: D-lactate dehydrogenase (EC: 1.1.1.28) was found in all *Desulfitobacterium* species except *D. metallireducens* DSM 15288^T^.

The genome of *Desulfitobacterium elongatum* NIT-TF6 contains genes encoding dehydrogenases for hydrogen (EC: 1.12.1.2), pyruvate (EC 2.7.1.40), malate (EC 1.1.1.38 and 1.1.1.40), and lactate. Fermentation of sugar and organic acids does not occur due to the absence of glucose transporter and acylphosphatase (EC: 3.6.1.7) for the substrate-level phosphorylation, respectively. The uniqueness in the genome for strain NIT-TF6 is the presence of genes coding L-lactate dehydrogenase (L-LDH, EC 1.1.1.27) in addition to another different type of L-lactate dehydrogenase complex (LldEFG, no EC number) [30]. Meanwhile, all other *Desulfitobacterium* species have only the gene of LldEFG (Figure 4). D-lactate dehydrogenase (D-LDH, EC 1.1.1.28) is not only present in *D. metalllireducens* DSM 15288^T^ among *Desulfitobacterium* species. Various bacterial species can produce D-lactate or both D- and L-lactates. However, major enzymes transforming lactate, such as LldEFG and LDH, have isometric specificity with some exceptions [30]. The presence of two types of L-lactate dehydrogenase and D-LDH in strain NIT-TF6 increases the availability of lactate for energy conversion and assimilation.

Due to the strain NIT-TF6 not having fermentative growth, oxidative phosphorylation is the only energy conservation mechanism in the strain. The NAD(P)Hs released from e^−^donors initiate the electron transport chain, which results in proton motive force for ATP synthesis. The electron transport can be terminated via thiosulfate reduction to sulfite and sulfide, coupling the oxidization of menaquinol by thiosulfate reductase (EC 1.8.5.5). A set of genes for dissimilative sulfite reduction (dsrABC) is present in the genome of strain NIT-TF6 despite its inability to grow on sulfite as well as on all *Desulfitobacterium* that grow on sulfite. The reason why there is no sulfite reduction in the strain has not been clarified in this study. The gene coding ferredoxin-nitrite reductase (EC 1.7.7.1) was present, suggesting that nitrite can be transformed into ammonia. Regarding assimilation, carbon dioxide can be fixed with formate to acetate via the reductive acetyl-coA pathway. This pathway is commonly conserved in all *Desulfitobacterium* spp. and *D. restrictus* DSM 9455^T^.

### 3.6. Unique Menaquinone Synthesis Pathway Based on the Genome

**Figure 5:**
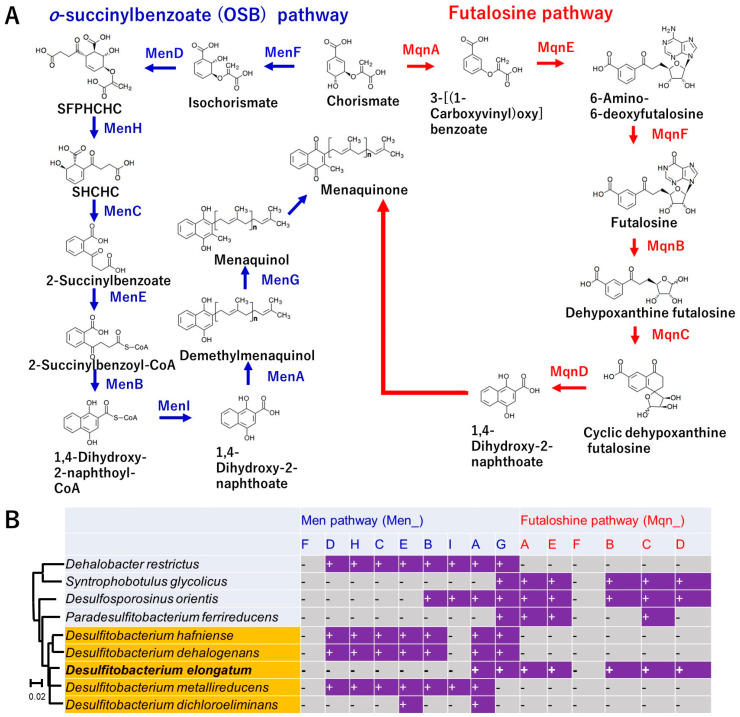
Comparison of genes coding enzymes involved in the menaquinone synthesis among *Desulfitobacteriaceae*. Panel (**A**) indicates the names of genes with coding enzymes involved in menaquinone synthesis. Panel (**B**) indicates the list of genes present in strains of *Desulfitobacteriaceae*. The symbols “+” and “-” indicate the presence and absence of the genes in their genomes, respectively.

The uniqueness in the genome contents and particularly in strain NIT-TF6 among *Desulfitobacterium* spp. in the assimilation was the menaquinone synthesis pathway. It was only the strain NIT-TF6 that had the most essential genes for the futalosine pathway (*mqnABCDE*) for menaquinone synthesis. However, all the other *Desulfitobacterium* mainly had the o-succinylbenzoate (OSB) pathway for synthesis (Figure 5). Besides *Desulfitobacterium*, *Dehalobacter restrictus* has OSB pathway-related genes. Meanwhile, *Syntrophobotulus glycolicus, Desulfosporosinus orientis*, and *Paradesulfitobacterium ferrireducens* had futalosine pathway-related genes. *D. orientis* also had partial genes for the OSB pathway. The absence of *menF* and *mqnF* in all strains was likely underestimated. This is because the identification system did not identify the gene as the target when the gene was most relevant to other genes, including showing sufficient e-values with the target. The differentiation of menaquinone biosynthesis has been reported to be an adaptation to the great oxygenation event. The futalosine pathway is the more ancient route, mostly found in aerobes and facultative anaerobes. Meanwhile, the OSB pathway is only found in aerobic, facultative anaerobic, and anaerobic prokaryotes. Differentiation in menaquinone synthesis among the *Desulfitobacteriaceae* family likely happened because of differentiation in genera concomitantly within the family.

### 3.7. The RdhA Gene Cluster and Mechanism for the Inability to Dechlorinate Chloroethene

Genomic analysis showed the presence of the catalytic subunit of reductive dehalogenase (RdhA)—coding gene (rdhA)—in *Desulfitobacterium elongatum* NIT-TF6, which is closely related to pceA from *Desulfitobacterium elongatum* and shares 94–95% nucleotide identity with *D. restrictus* strains DSM 9455 and TeCB1, which have previously demonstrated the ability to reductively dechlorinate tetrachloroethene (PCE) to 1,2-*cis*-DCE (dichloroethane) and 1, 2, 4, 5-tetrachlorobenzene to 1,3- and 1,4-dichlorobenzene [7,31]. Besides rdh*A*, the membrane-bound subunit of reductive dehalogenase (rdh*B*) in NIT-TF6 had 98% identity with that of “*D. dichloroeliminans* DCA1”, and another membrane-bound subunit (rdhC) sharing 98% identity with *D. restrictus* and 96–97% identity with *D. hafniense* and “*D. dichloroeliminans*”. These reductive dehalogenase-related genes are present near a transposase-coding gene and are likely acquired through horizontal gene transfer as reported in other organohalide-respiring *Desulfitobacterium*.

**Figure 6:**
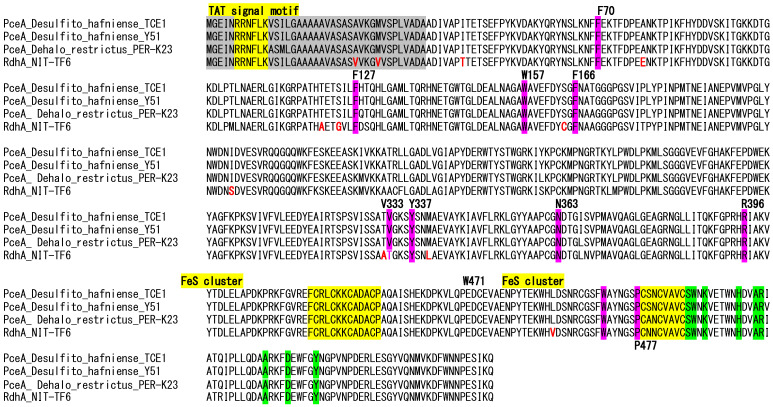
Amino acid sequences between strain NIT-TF6, *D. hafniense* DCB-2^T^, and *D. restrictus* DSM 9455^T^. Red color indicates the difference in amino acid found only in strain NIT-TF6. The yellow region indicates the conserved domain of the TAT signal and Fe-S clusters. Pink-colored residues are important residues of the active site, while green indicates those for menaquinol binding sites.

The presence of full sets of Rdh-related genes in the genome suggested the organohalide respiration capability of the strain NIT-TF6. However, the strain NIT-TF6 did not dechlorinate tetrachloroethene, trichloroethene, 1,2-dichloroethene, and 1,2-cis-dichloroethene. Comparing amino acid sequences among strain NIT-TF6, *Desulfitobacterium hafniense* DCB-2, and *D. restrictus* DSM 9455 showed that the most important amino acid residues of the active site were identical among the three strains, except for one replacement of V333 from a non-polar amino acid to thiamine of polar amino acid. The replacement potentially inactivated the RdhA to dechlorinate CEs. Figure 6 illustrates amino acid sequences of NIT-TF6 and its closest relatives.

### 3.8. Role of Strain NIT-TF6 in the TCE-Dechlorinating Culture

The genome suggests two potential roles for the *Desulfitobacterium elongatum* NIT-TF6 in supporting TCE dechlorination by *Dehalococcoides* in the microcosm: (1) participating in the dechlorination of chloroethenes in conjunction with *Dehalococcoides*, and (2) producing acetate through acetogenesis using formate and carbon dioxide. However, strain NIT-TF6 did not dechlorinate any chloroethenes or chloroethanes. Attempts to confirm the acetate formation from ^13^C-formate and ^13^C-carbonate were unsuccessful, as no significant increase in ^13^C-acetate was observed in cultures repeatedly transferred with ^13^C-labeled substrates. One potential role of strain NIT-TF6 could be the provision of H_2_ to *Dehalococcoides*. The strain was found to produce H_2_ from formate. However, this capability is not unique to *Desulfitobacterium* and is not a specialized trait of this genus. Therefore, the exact reason why *Desulfitobacterium* coexists with *Dehalococcoides* in the microcosm remains unclear based on the findings of this study.

## 4. Conclusions

Strain NIT-TF6, belonging to the genus *Desulfitobacterium*, was isolated from a TCE-dechlorinating microcosm using formate as an energy and carbon source. The exact reason for the coexistence of *Desulfitobacterium* with *Dehalococcoides* in the culture remains unclear based on physiological and genomic characterization. This is because of the apparent uniqueness in the genome of strain NIT-TF6, namely the presence of genes for L-LDH and the futalosine pathway of menaquinone synthesis. The strain exhibited 91.31–96.39% sequence similarity in the 16S rRNA gene to *D*. *hafniense* DCB-2^T^ and shared 15.9% dDDH and 71.88% ANI with its closest relative. As the unique genomic characteristics and these values are below the thresholds for species delineation, strain NIT-TF6 is proposed as the type strain of a novel species, *Desulfitobacterium elongatum* sp. nov., with the type strain NIT-TF6^T^.

### Description of Desulfitobacterium elongatum sp. nov.

*Desulfitobacterium elongatum* (e.lon.ga’tum. L. neut. part. adj. elongatum, elongated) is gram-positive, spore-forming, straight rod-shaped, and strictly anaerobic. Thiosulfate and Fe (III) citrate were used as electron acceptors when pyruvate was used as electron donor. H_2_+acetate, pyruvate, lactate, and malate were used as electron donors when thiosulfate was used as electron acceptors. The strain did not grow on formate, acetate, citrate, fumarate, propionate, butyrate, methanol, ethanol, propanol, and benzoate in thiosulfate respiration. Sulfate, sulfite, nitrate, nitrite, and air were not reduced. The type strain, NIT-TF6^T^ was isolated from Arako river sediment in Nagoya, Japan. The DNA G+C content of the type strain is 43.2 mol%, which was achieved based on the complete genome sequence (AP038772).

## Figures and Tables

**Table 1:** Useful characteristics for differentiating strain NIT-TF6 from closely related members of the genus *Desulfitobacterium*.

	1	2	3	4	5
Size (µm)	1.6 − 6 × 0.25 − 0.5	2.5 − 4 × 0.5 − 0.7	2 − 5 × 0.5 − 0.7	3.3 − 6 × 0.6 − 0.7	2 − 5 × 0.5
Spores	+	−	ND	+	−
Cell morphology	Straight Rod	Straight or curved rod	Curved rod	Straight rod	Curved rod
e^−^ donor for thiosulfate respiration					
H_2_+acetate	+	ND	+	ND	−
Formate	−	+	+	−	+
Acetate	−	−	−	ND	−
Citrate	−	ND	ND	ND	−
Fumarate	−	ND	ND	ND	ND
Malate	+	ND	ND	ND	+
Pyruvate	+	+	ND	+	−
Lactate	+	+	+	+	+
Propionate	−	−	ND	−	−
Butyrate	−	+	ND	−	+
Methanol	−	−	ND	ND	ND
Ethanol	−	+	ND	ND	+
Propanol	−	ND	ND	ND	−
Benzoate	−	ND	ND	−	−
e^−^ acceptors for pyruvate oxidization					
Thiosulfate	+	+	+	+	+
Sulfate	−	−	−	−	−
Sulfite	−	+	+	+	−
Nitrate	−	+	+	+	−
Nitrite	−	ND	−	ND	−
Fe(III)-citrate	+	ND	ND	ND	+
O_2_ (air)	−	−	ND	ND	−

Strains are designated as 1, 2, 3, 4, and 5. 1: *Desulfitobacterium elongatum* NIT-TF6; 2: *D. dehalogenans* ATCC 51507^T^ [2]; 3: “*D*. *dichloroeliminans*” DCA1 [4]; 4: *D. hafniense* DCB-2^T^ [1]; 5: *D. metallireducens* DSM 15288^T^ [3]. +, used; −, not used; ND, not denoted; T, Type strain.

**Table 2:** Genomic features of strain NIT-TF6 with other *Desulfitobacterium* valid strains. Column headers 1, 2, 3, 4, and 5 indicate the following: 1: *Desulfitobacterium elongatum* NIT-TF6; 2: *D. dehalogenans* ATCC 51507^T^ [2]; 3: “*D*. *dichloroeliminans*” DCA1 [4]; 4: *D. hafniense* DCB-2^T^ [1]; 5: *D. metallireducens* DSM 15288^T^ [3].

Features	1	2	3	4	5
Size (Mb)	4.8	4.3	3.6	5.2	3.1
GC (%)	43.2	45	44.2	47.5	41.9
CDSs	4655	4403	3544	5247	3138
16S rRNAs	9	6	6	5	8

## Data Availability

The original contributions presented in this study are included in the article/Supplementary Materials. Further inquiries can be directed to the corresponding author.

## Supplementary Materials

The following supporting information can be downloaded at: https://www.mdpi.com/article/10.3390/microorganisms13081863/s1, Table S1. Organic pollutants dechlorination activity for the strain NIT-TF6; Figure S1: Phylogenomic tree based on TYGS results showing relationship between strain NIT-TF6 and its related cluster of (a) Desulfitobacteriacea family (restricted query to the genomes). Leaf labels are annotated by affiliation to species and subspecies clusters, genomic G+C content, δ values, overall genome sequence length, number of proteins and kind of strain.
