# Supplementary material for: Desulfitobacterium elongatum sp. nov. NIT-TF6 Isolated from Trichloroethene-Dechlorinating Culture with Formate
Source: Microorganisms. 2025 Aug 9;13(8):1863. doi: 10.3390/microorganisms13081863 (PMC12388241; doi:10.3390/microorganisms13081863)
Supplement: Supplementary file 1 [file microorganisms-13-01863-s001.zip › Figure S1.pdf]

Supplementary material

Desulfitobacterium elongatum sp. nov. NIT-TF6 Isolated from Trichloroethene-Dechlorinating Culture with Formate

Udaratta Bhattacharjee [1,2,†], Ryuya Tomita [1], Li Xie [3] and Naoko Yoshida [1,4,*,†]

[1] Department of Civil and Environmental Engineering, Nagoya Institute of Technology (Nitech), Gokiso-Cho, Showa-Ku, Nagoya 466-8555, Japan
[2] Department of Biotechnology, School of Bio Engineering, SRM-Institute of Science and Technology, Kattankulathur, Chennai 603203, India
[3] State Key Laboratory of Lake and Watershed Science for Water Security, Nanjing Institute of Geography and Limnology, Chinese Academy of Sciences, Nanjing 211135, China
[4] Department of Civil Engineering, Graduate School of Engineering, Nagoya University, Nagoya 464-8603, Japan
* Correspondence: yoshida.naoko.r0@f.mail.nagoya-u.ac.jp; Tel.: +81-52-735-5437
† These authors have contributed equally to this work.

Supplementary Figure S1

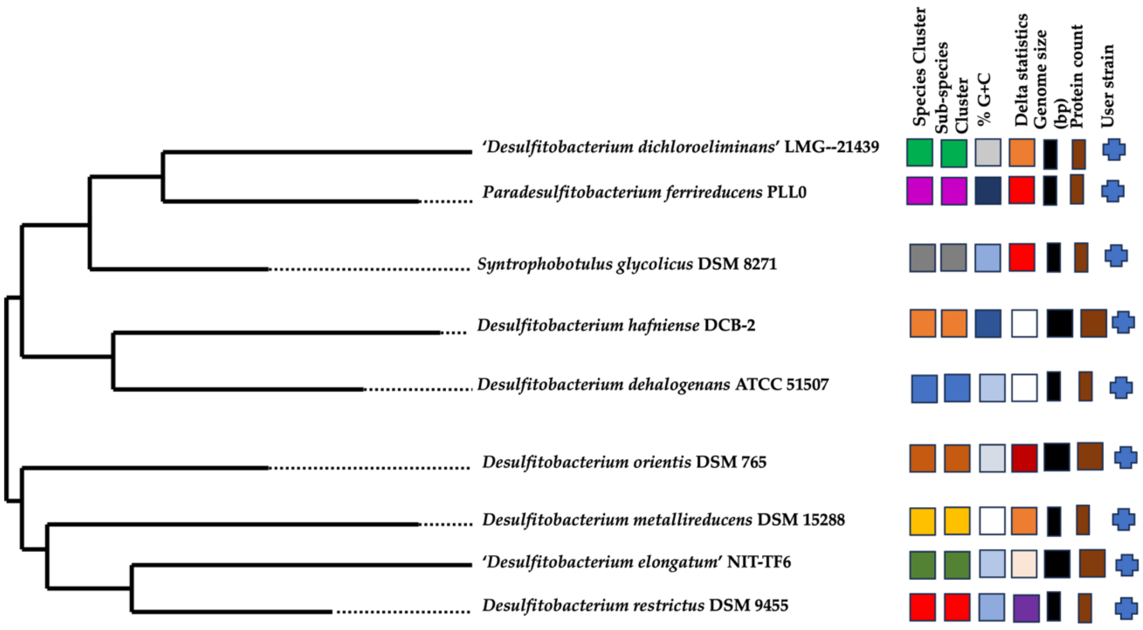

Figure S1. Phylogenomic tree based on TYGS results showing relationship between strain NIT-TF6 and its related cluster of (a) *Desulfitobacteriacea* family (restricted query to the genomes). Leaf labels are annotated by affiliation to species and subspecies clusters, genomic G+C content, δ values, overall genome sequence length, number of proteins and kind of strain.
